# Supplementary material for: Tead1a Initiates Transcriptional Priming Through the TEAD1a/YAP‐Notch1‐Spi1/Cebpα Axis to Promote Neutrophil Fate
Source: Adv Sci (Weinh). 2025 Aug 28;12(41):e05441. doi: 10.1002/advs.202505441 (PMC12591127; doi:10.1002/advs.202505441)
Supplement: Supplementary file 4 — Supplementary Dataset [file ADVS-12-e05441-s004.zip › 3-2.zip!/3-2/02-2.cluster/cluster5/Enrich/ego_all.pdf]

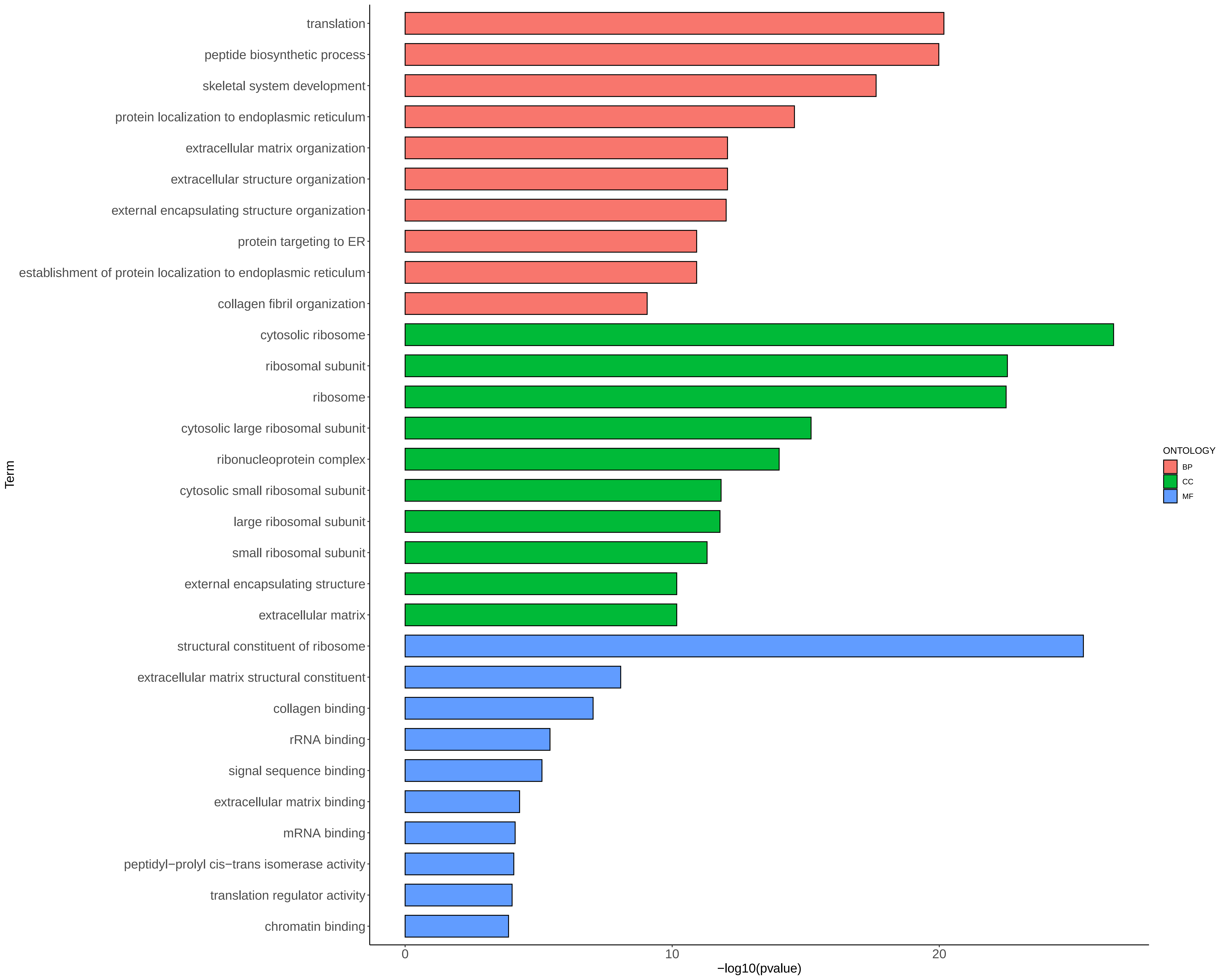
translation
peptide biosynthetic process
skeletal system development
protein localization to endoplasmic reticulum
extracellular matrix organization
extracellular structure organization
external encapsulating structure organization
protein targeting to ER
establishment of protein localization to endoplasmic reticulum
collagen fibril organization
cytosolic ribosome
ribosomal subunit
ribosome
cytosolic large ribosomal subunit
ribonucleoprotein complex
cytosolic small ribosomal subunit
large ribosomal subunit
small ribosomal subunit
external encapsulating structure
extracellular matrix
structural constituent of ribosome
extracellular matrix structural constituent
collagen binding
rRNA binding
signal sequence binding
extracellular matrix binding
mRNA binding
peptidyl−prolyl cis−trans isomerase activity
translation regulator activity
chromatin binding
Term
0
10
20
−log10(pvalue)
ONTOLOGY
BP
CC
MF
